# Burnout, depression, and medication errors among family physicians

**DOI:** 10.1038/s41598-025-29450-z

**Published:** 2025-12-09

**Authors:** Dina Fawzy Abd Elsadek, Amira B. Kassem, Fayek ElKhwsky, Iman El Sayed

**Affiliations:** 1 Department of Biomedical Informatics and Medical Statistics, Medical Research Institute, Alexandria University, Alexandria, Egypt; 2 Department of Clinical Pharmacy and Pharmacy Practice, Faculty of Pharmacy, Damanhur University, Damanhur, Egypt

**Keywords:** Burnout, Depression, Prescribing errors, Medication error, Physicians, Health services, Occupational health

## Abstract

Physician burnout has been recognized as a global healthcare threat. Burnout adversely affects doctors’ well-being, patient care costs, and quality. The impact of burnout on medication error is debatable since most literature relied on self-reporting, whereas studies utilizing objective measures reported contrary results. Our study investigates physicians’ burnout prevalence and association with detected prescribing errors in ambulatory settings. A cross-sectional study was conducted in 25 primary healthcare centers in Alexandria, Egypt. The Professional Fulfillment Index and Patient Health Questionnaire-9 were used to screen for burnout and depression among physicians. Clinical pharmacists independently reviewed 50 prescriptions per physician to identify decision and process prescribing errors. The prescribing errors were assessed by reviewing drug suitability for the patient’s condition according to best practice guidelines. Risk variables for prescribing errors were evaluated using multivariable negative binomial analysis. Researchers collected prescriptions from 118 doctors out of 184 who completed the survey. Almost half of the responders suffered from burnout and depression symptoms (42.39% and 42.93%), while most of them reported low personal professional fulfillment (89.13%). Of 14,121 medications evaluated, 6543 prescribing errors were detected (46.34%), with only 0.11% of them potentially serious. The most prevalent prescribing error types were wrong dosage regimens, incomplete patient information, and missing drug instructions. Analgesics, systemic antihistaminics, and antibacterials were the most misprescribed ATC drug categories. Prescribing errors had no significant association with burnout, professional fulfillment, depression, or thoughts of quitting among doctors. Males, consultants, and rural work settings physicians were more prone to commit ordering errors. Prescribing errors were prevalent in primary care settings, but they were not significantly linked to physicians’ burnout, professional fulfillment, or depression. The burden of burnout and depression among physicians, as well as the complexity of evaluating their impact on actual patient safety, highlights the need for further research and tailored interventions.

**Supplementary Information:**

The online version contains supplementary material available at 10.1038/s41598-025-29450-z.

## Introduction

The 11th revision of the International Classification of Diseases (ICD-11) included burnout as an occupational syndrome adversely affecting health status or healthcare services and arising from unmanaged prolonged work-related stress^1^. Three characteristics define burnout: “feelings of energy depletion or exhaustion; increased mental distance from one’s job, or feelings of negativism or cynicism related to one’s job; and reduced professional efficacy”^1^.

Physicians faced a 41% higher risk of burnout than the general population^2^. Burnout was recognized as a global healthcare problem since it affected more than 50% of clinicians in the United States, with similar or higher prevalence recorded in other countries^3^. In Egypt, burnout prevalence ranged from 22 to 77% among physicians, with a pooled rate of 60.59%^4^. Front-line specialties such as emergency medicine and family medicine are more prone to burnout^5^.

Burnout was linked to workplace stressors, including electronic medical records, long and conflicting work hours, insufficient compensation, work-life imbalance, excessive work demands, unsupportive coworkers and leadership, and limited control over the work environment^6^. Disparities in burnout susceptibility among physicians may be attributed to personal traits such as ineffective coping mechanisms, doubt, perfectionism, neuroticism, young age, relationship status, female gender, partner occupation, and immature children^7^. Despite not being categorized as a medical condition, burnout has gained much attention in recent years due to its multifaceted consequences on the patient and physicians themselves^6^.

A primary cause of avoidable injury in health care is drug safety incidents^8^. Medical errors ranked as the third most frequent cause of death in the United States^9^. Among healthcare recipients, 1 out of 30 suffered an avoidable medication-related injury; 26% of these are classified as serious or life-threatening, and 58% occurred during the prescribing stage^10^. Prescription errors were the most frequently reported medication errors in Egypt (54%)^11^. An estimated annual cost of US$ 528.4 billion was related to suboptimal medicine use-related morbidity and mortality in the U.S., representing up to 16% of all healthcare costs in the country in 2016^12^.

Despite the cumulative data that links burnout with adverse patient outcomes, current research has primarily relied on physician and patient self-reports of errors rather than objective measures of patient outcomes^13,14^. In a systematic review focusing on physician burnout and its impact on patient outcomes, an analysis of 14 studies assessing physicians’ self-perceptions revealed a significant association between burnout and suboptimal care or medical errors. However, an objective evaluation of clinical charts via 5 studies failed to detect any relationships^15^. Due to self-criticism that can be exaggerated by burnout, physicians’ self-reported data on their performance is less reliable^16^. In a cross-sectional study including 7395 surgical residents in the United States, burnt-out residents were three times more likely to self-report harmful medical errors; however, there was no difference in objectively evaluated postoperative outcomes for surgical patients^17^. Additionally, depression was linked to both physician burnout and medical errors, except for a few studies that reported the association of objectively measured medical errors with depression in contrast to burnout^18–21^.

In 2019, the World Health Organization (WHO) reported minimal progress in the global reduction of patient harm over the past 15 years^22^. They recommended a better understanding of intangible aspects influencing patient safety and their implication in policy and practice, particularly in primary care, where half of the global burden of patient harm occurs^22^. Over half (53%) of the avoidable medication-related harm worldwide occurred at the prescribing phase, and the percentage increased to 80% in low- and middle-income countries^23^. Because physicians’ burnout influences healthcare quality, it’s critical to comprehend how burnout affects objectively measured patient outcomes. Therefore, this study aimed to investigate the prevalence of burnout and medication prescribing errors among primary healthcare physicians and the association between them after adjusting for depression.

## Methods

### Study design and participants

This was a multi-centric cross-sectional study of family physicians in 25 primary health care (PHC) units in Alexandria between March 1, 2022, and February 28, 2023. Alexandria governorate takes up the ninth rank in descending population distribution of Egyptian governorates in 2024^24^. In Egypt, Family Health Units (FHU) are the initial stage of comprehensive care for the person, family, and community^25^. In 2021, family health units represented 90.5% of primary health care settings in Alexandria^26^. FHU has a specific list of 110 essential medications and vaccines.

All participants signed written informed consent. The research was approved by the ethical review board of the Medical Research Institute and the Ministry of Health and Population, and it adhered to the Declaration of Helsinki’s ethical guidelines. All the PHC clinicians in the family care units at the time of data collection were eligible for voluntary participation.

### Sample size

According to a pilot of available registers of family physicians, the total target population was approximately 500 family physicians working in Alexandria PHC units. At a 95% confidence interval and 5% precision limit, a minimum sample size of 181 physicians was calculated based on the estimated burnout prevalence of 24.3% from a previous research in a Saudi Arabian primary care setting^27^. For the second outcome, the proportion of burnout in physicians who reported errors was 77.6% versus 51.5% in those who did not report errors^28^. A sample size of 118 participants can confirm this effect with 80% power and a 5% significance level by using Epi Info 7^29^.

### Study measures

The physicians received a self-administered survey categorized into 3 categories: participants’ characteristics, the Professional Fulfillment Index (PFI), and the Patient Health Questionnaire-9 (PHQ-9) to assess burnout and depression^30,31^. We employed the Questionnaires’ English version.

#### Participants’ characteristics

Basic demographics included age, sex, marital status, number of children, partner work, ride time to work, exercise frequency, history of anxiety or depression, and chronic diseases. Participants’ occupational details included practice years, certificates, occupation, employer status, practice setting, managerial status, number of patients seen per shift, weekend work frequency, and intention to quit the practice.

#### Burnout and professional fulfillment measurement

The validated 16-item PFI survey uses a 5-point Likert scale to assess professional fulfillment (6 items) and the overall burnout scale (10 items)^31^. Response options for professional fulfillment items varied from “not at all true” to "completely true," while burnout scale alternatives ranged from “not at all” to “extremely”^31^. Permission was acquired for using the questionnaire.

The scale scores are derived by averaging all items’ scores on each related scale and then multiplying by 2.5 to scale from 0 to 10. Professional fulfillment and overall burnout scale cut points are 7.5 and 3.325, respectively^32^. Higher scores indicate favorable outcomes in professional fulfillment and negative experiences in burnout^32^.

#### Depression measurement

The PHQ-9 is a validated, self-administered tool for identifying, categorizing, and monitoring depressive symptoms^30^. The frequency of depressed symptoms was evaluated during the preceding two weeks using four responses ranging from 0 "not at all" to 3 "nearly every day", with a total score between 0 and 27^30^. A score of 10 or higher was considered indicative of depressive symptoms in participants^33^.

#### Medication prescribing errors

A prescribing error occurs when the physician’s ordering decision or writing process results in a lower probability of efficacy or a higher risk of adverse effects of treatment^34^. Based on the Ghaleb et al. and Dean et al. scenarios, the prescribing errors were classified with adjustments to local clinical reporting forms and the study’s outpatient setting:

1. Decision-ordering error options included drugs with no valid indication, therapeutic duplication, indication without medication, drug contraindication, drug interaction without proper management, wrong dosing regimen, wrong duration, wrong dosage form, or wrong dilution instruction^34,35^.

A wrong dose was identified as exceeding 20% of the recommended dose for a drug of wide therapeutic index, and prescribing when needed (PRN) without specifying the minimal dose interval^36,37^.

2. Process ordering error options included the wrong patient, incomplete or wrong required patient information, wrong drug name, illegible handwriting, wrong strength, wrong decimal point, incomplete prescribing instructions, repetition, or unapproved abbreviations^34,35^.

The patient’s name, age, gender, diagnosis, and child weight (if appropriate) are the basic required patient information in the prescription^36^.

**Medication prescribing errors detection process**

In Egyptian PHC, prescriptions are handwritten without adopting standard coding for diseases or electronic prescribing systems. The Prescription has 1 or more prescribed medications. Researchers collected the medication data by reviewing the first 50 available prescriptions for each physician in the primary health care units’ pharmacies. Two experienced clinical pharmacists independently checked the appropriateness of drug therapy for the patient’s condition according to best practice guidelines.

Evidence-based sources included Lexicomp, UpToDate, British National Formulary (BNF), Centers for Disease Control (CDC), National Institute for Healthcare and Excellence (NICE), Drugs.com, Medscape, disease-specific guidelines, and others for identifying ordering errors.

The pharmacists recorded a description of the event, the drug’s category, the classification of ordering error types, and the categorization of the potential harm of each event. The classification of the potential harm associated with ordering errors varied from none to the potential of severe harm^38^. The pharmacist reviewers’ disagreement was settled by consensus via discussion. The drugs were classified using the second level of the WHO Anatomical Therapeutic Chemical (ATC) categorization system^39^.

### Statistical analyses

The ordering error rate (%) was obtained by dividing total errors by the number of prescribed medications and then multiplying by 100^40^. Multiple errors in the same medication were counted separately.

Quantitative data were presented as a median plus interquartile range, while qualitative data were summarized as frequencies and percentages. The Kolmogorov–Smirnov test detected the continuous data’s deviation from normality^41^. Due to the overdispersion in the poisson models, negative binomial analysis was used to identify parameters related to prescribing error rates, with the number of prescribed items as an offset in the models. The multivariate regression model included all variables with a P value <0.05 in the bivariate analysis. Data were analyzed using Statistical Package for the Social Sciences (SPSS version 28) and R Software, with a significance level set at *p* < 0.05^42,43^.

## Results

### Characteristics of the respondents

**Fig. 1:**
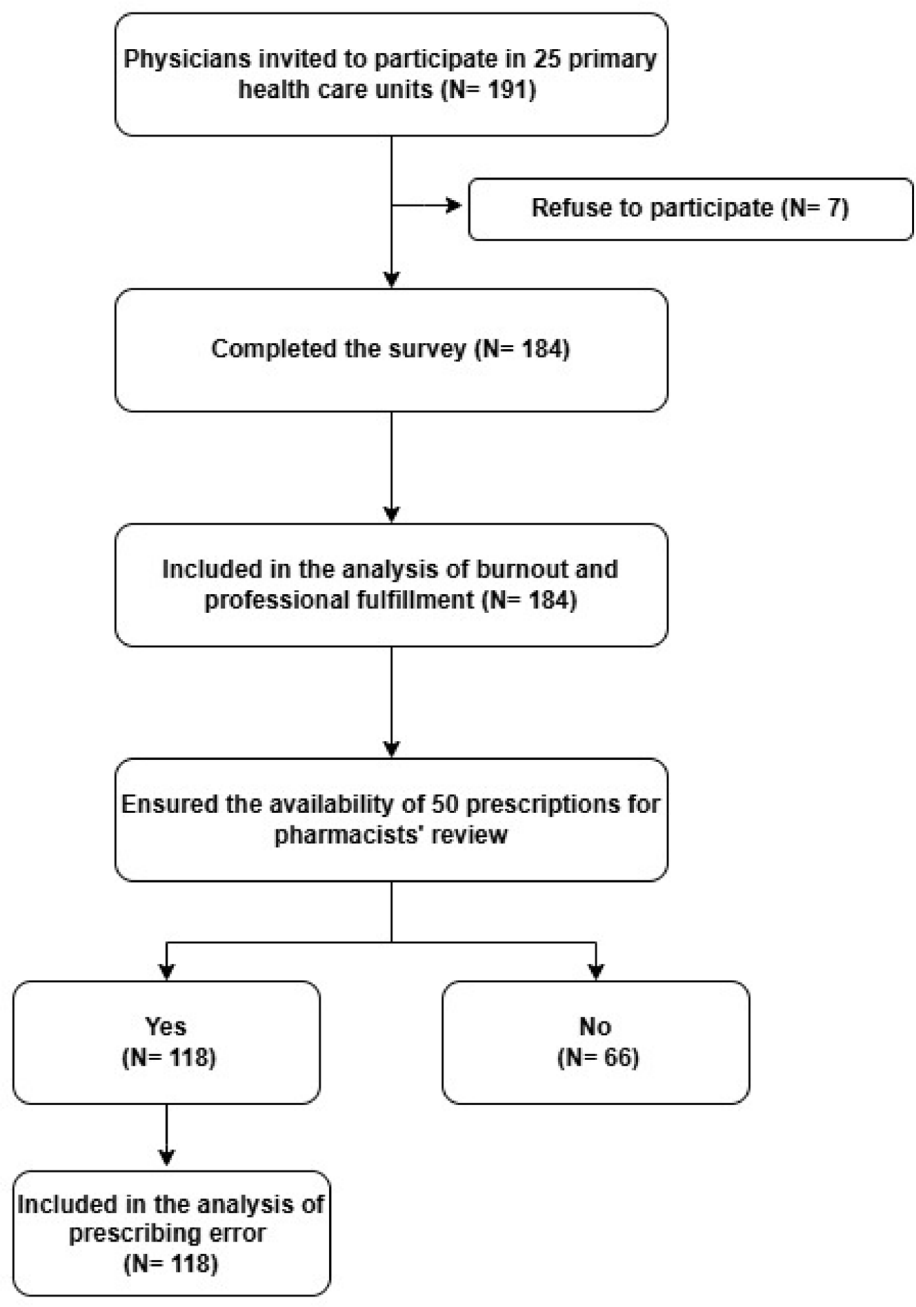
Flowchart of data collection and analysis.

The study included 184 family physicians (Fig. 1). The median age of the participants was 45 (interquartile range [IQR] 38.00–55.00) (Table 1). Most participants were females (94.02%), married (81.52%), and parents (86.41%). Less than half of the participants (44.57%) had at least one comorbidity, while 19.02% of the physicians reported a prior anxiety or depression diagnosis. Two-thirds (71.74%) of the respondents were sedentary, and only 6.52% exercised daily.

Most responders were family physicians (66.85%), full-time employees (93.48%), and non-managers (83.15%), with a median of 20 practice years (IQR 11.00–30.00). About one-quarter of respondents had only a bachelor’s degree, 36.41% completed a postgraduate diploma, and 40.22% earned master’s or higher degrees. The median number of patients examined per shift was 30, with an IQR of 20–40.

**Table 1:** Characteristics of the participants in primary health care centers in Alexandria, Egypt, 2022–2023 (n = 184).

Personal characteristics	No. (%) or Mdn (IQR)	Professional characteristics	No. (%) or Mdn (IQR)
**Gender**		**Occupation**	
Male	11 (5.98)	Family physician	123 (66.85)
Female	173 (94.02)	Specialist	61 (33.15)
**Age in years**, Mdn (IQR)	45.00 (38.00,55.00)	**Practice Location**	
**Marital status**		Urban	148 (80.43)
Single^a^	34 (18.48)	Rural	36 (19.57)
Married	150 (81.52)	**Employment status**	
**Partner works outside the home**		Part-time	12(6.52)
Yes	115 (62.50)	Full time	172 (93.48)
No	36 (19.57)	**Highest degree**	
**Working partner profession**		Bachelor	43 (23.37)
Physician	46 (25.00)	Diploma	67 (36.41)
Health care provider, non-physician	14 (7.61)	Master’s degree and higher	74 (40.22)
Non-health care	53 (28.80)	**Years in practice,** Mdn (IQR)	20.00 (11.00,30.00)
**Having children **		**Weekend work **	
Yes	159 (86.41)	˂ 2 / month	138 (75.00)
No	25 (13.59)	≥2 / month	46 (25.00)
**Children number**, Mdn (IQR)	2.00 (2.00,3.00)	**Ride time,** Mdn (IQR)	30.00 (20.00,45.00)
**Chronic disease**		**Management position**	
Yes	82 (44.57)	Yes	31 (16.85)
No	102 (55.43)	No	153 (83.15)
**Previous depression or anxiety diagnosis**		**Patients no. /shift,** Mdn (IQR)	30.00 (20.00,40.00)
Yes	35 (19.02)		
No	149 (80.98)		
**Physical exercise frequency**			
Monthly or less	132 (71.74)		
Weekly	40 (21.74)		
Daily	12 (6.52)		

Values are presented as No. (%) unless otherwise stated. Mdn, median; IQR, interquartile range. ^a^ Single marital status included single, separated, divorced, and widowed.

#### Physicians’ thoughts of abandoning the profession

In the previous 12 months, over 1 in every 3 participants frequently considered leaving their profession (34.78%), 33.15% of respondents thought about abandoning their profession occasionally, and 32.07% rarely or never.

#### Prevalence of burnout, professional fulfillment, and depression in the study’s sample

**Fig. 2:**
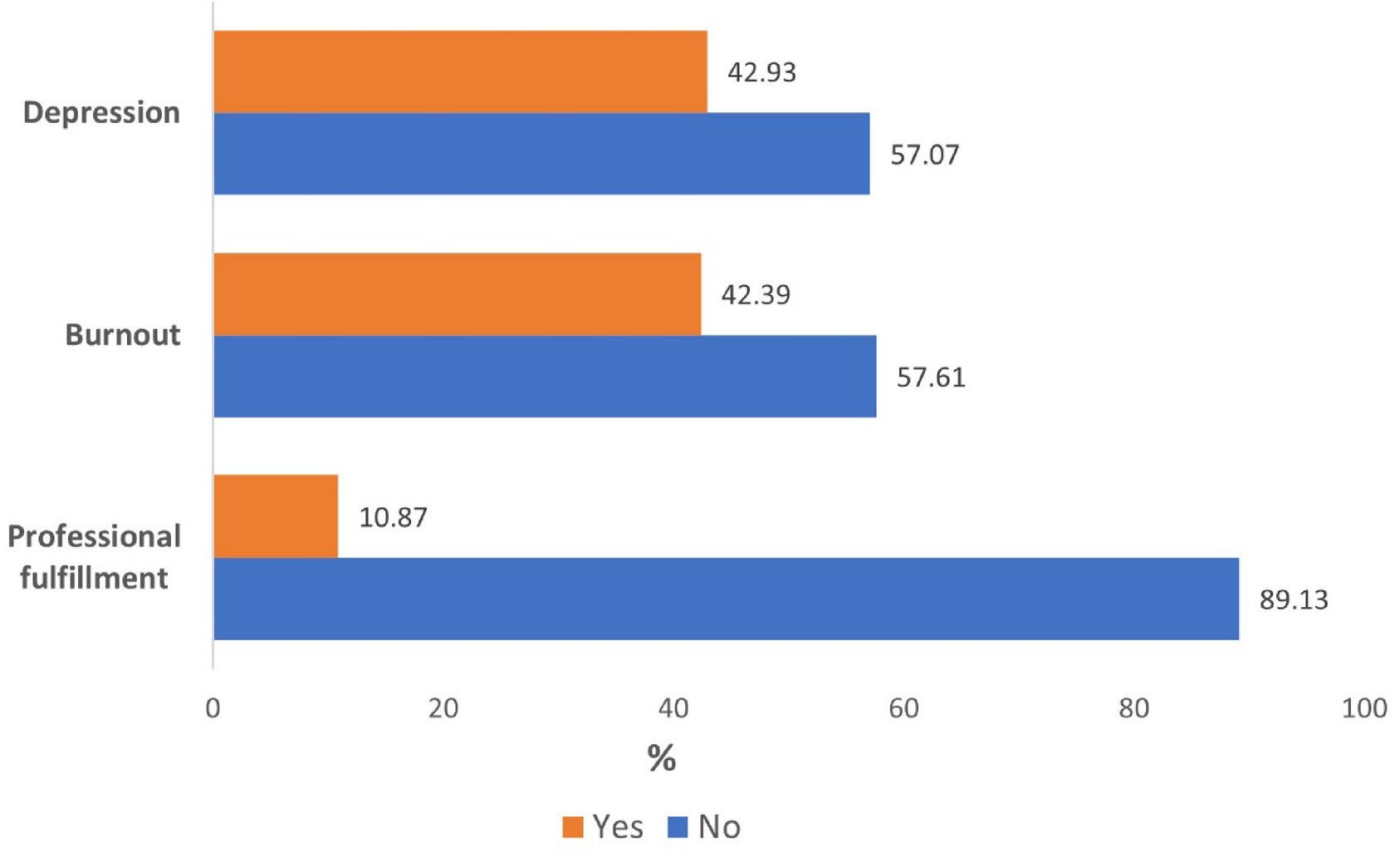
Prevalence of burnout, professional fulfillment, and depression among the study’s participants in primary health care centers in Alexandria, Egypt, 2022–2023.

About half of the participants were depression-symptom-free (57.07%), while 78 physicians (42.39%) met the criteria for burnout, and 164 (89.13%) had a low personal professional fulfillment score (Fig. 2).

### Types and prevalence of prescribing errors

A total of 5900 prescriptions for 118 family physicians were analyzed. The review of 14,121 prescribed medications revealed 6543 prescribing errors (PE) (46.34%). Errors in the prescribing decision-making occurred more frequently than in the process (63.12% vs. 36.88%). The wrong dose regimen was the most prevalent type of error (53.68%), followed by incomplete patient details (24.35%) and incomplete prescribing instructions (12.52%) (Table 2).

**Table 2:** The types and frequency (%) of prescribing errors at family health units in Alexandria, Egypt, 2022–2023.

Types of error	No	%
1. Decision ordering error (n = 4130, 63.12%)		
Wrong dose regimen (Dose/ Frequency)	3512	53.68
Drug interaction	244	3.73
Drug without indication	197	3.01
Wrong duration	98	1.50
Indication without drug	50	0.76
Drug contraindication	17	0.26
Therapeutic duplication	8	0.12
Wrong dilution instructions	3	0.05
Wrong route of administration	1	0.02
2. Process ordering error (n = 2413, 36.88%)		
Incomplete or wrong patient information	1593	24.35
Incomplete prescribing instructions	819	12.52
Wrong drug Name	1	0.02

The percentages represent the proportion of the total number of prescribing errors.

### Potential Harm of Prescribing Errors

**Fig. 3:**
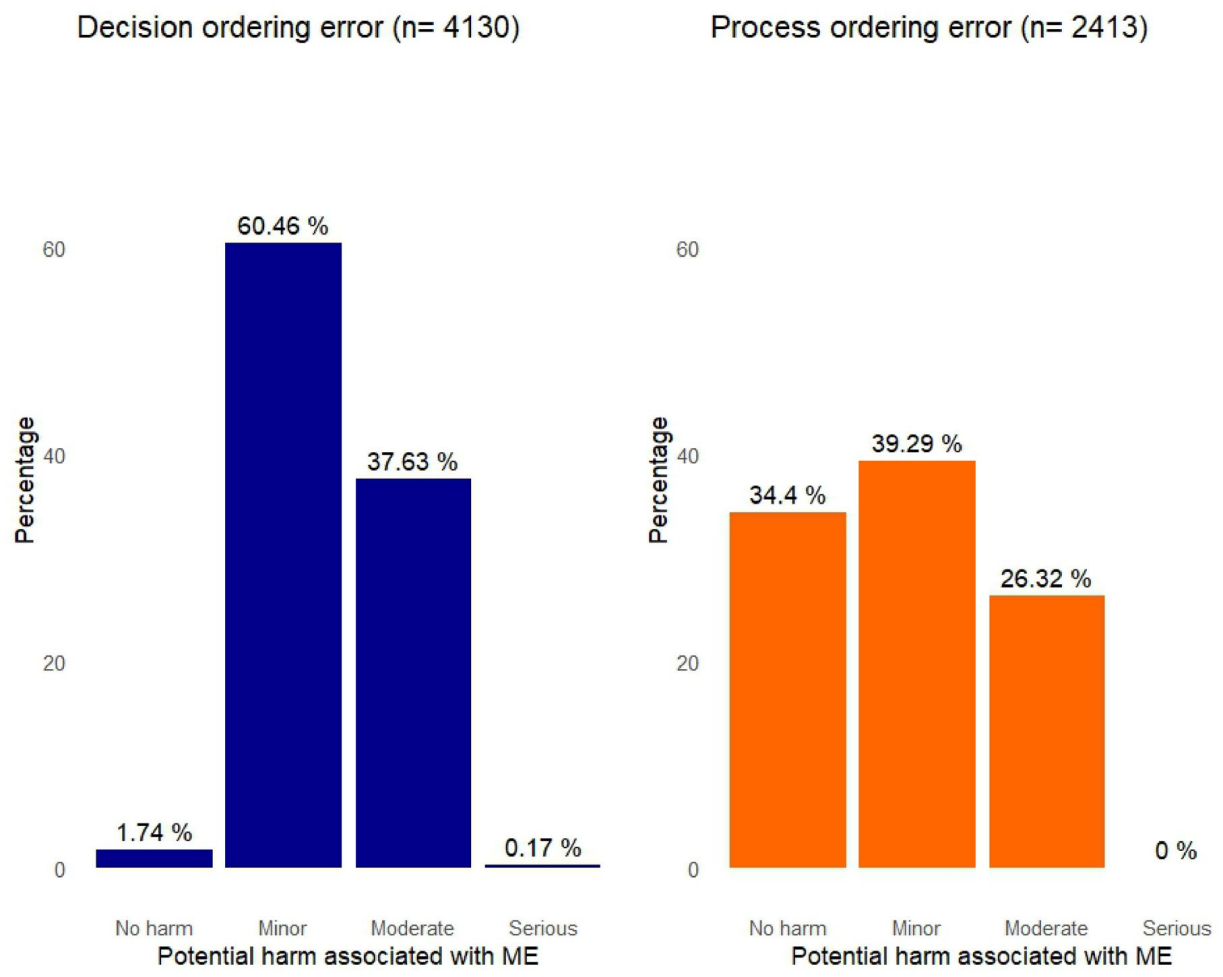
Potential severity of prescribing errors based on the error types in primary health care units, Alexandria, Egypt, 2022–2023. ME, medication error.

Half of the prescribed errors posed minor potential harm to patients, 33.46% moderate severity, and 13.79% no potential risks. Only 0.11% of errors were serious, and there was no potential for severe harm. The category of ordering errors was associated with the potential severity of errors (*P* ˂0.001) (Fig. 3). Decision and process ordering errors were primarily responsible for minor potential harm (60.46% vs. 39.29%), and 37.6% of decision prescribing errors were moderate in severity, compared to 26.32% of process errors. Only 0.17% of decision errors had a potentially serious impact, while process errors did not contribute to serious concerns. Examples of prescribing errors and potential harm are provided in Supplementary Table S1.

### The top 10 misprescribed drug categories

**Fig. 4:**
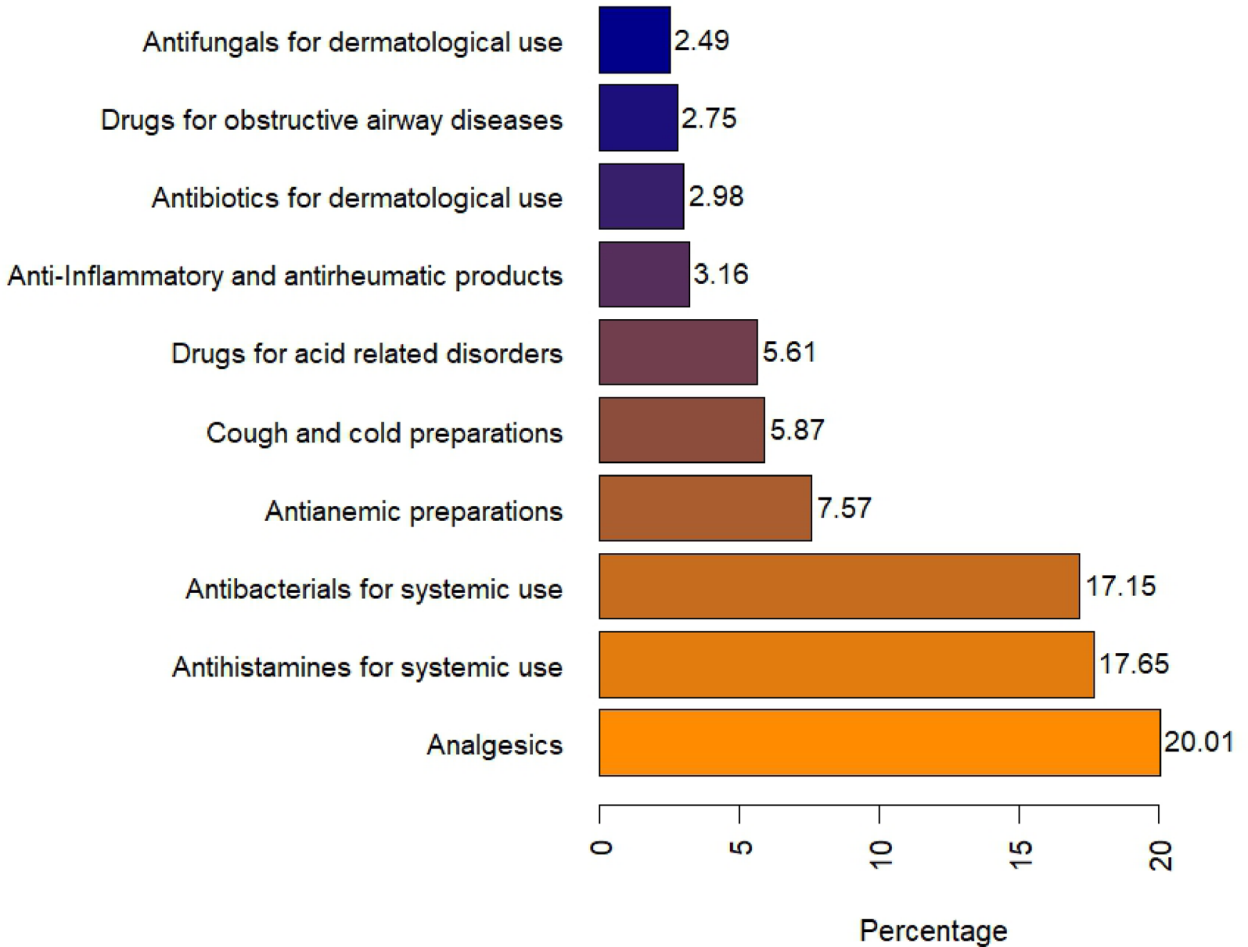
Top drug classes involved in the prescribing errors according to the anatomical therapeutic chemical classification system in primary health care units, Alexandria, Egypt, 2022–2023.

Based on the ATC classification system, Fig. 4 illustrates the top ten medication categories with prescribing errors. Analgesics, systematic antihistaminics, and systemic antibacterials were the most frequently misprescribed drug classes (20.01%, 17.65%, and 17.15%, respectively). Supplementary Table S2 indicates drug classification according to the ATC system.

### Factors associated with prescribing error rate

Table 3 illustrates the findings of the unadjusted and adjusted analysis of variables linked with the prescribing error rate. In the unadjusted analysis, the factors with a significant association included gender, chronic illness history, occupation, practice location, and occasional thoughts of leaving the profession.

**Table 3:** Negative binomial regression analysis of Participants’ Characteristics related to ordering error rates per 100 prescribed Items.

	Unadjusted rate ratio (95% CI)	*P*-value	Adjusted Rate ratio (95% CI)	*P*-value
**Male gender** († female)	1.51 (1.15–2.02)	0.005*	1.50 (1.13–2.01)	0.005*
**Age** in years	0.995 (0.99–1.00)	0.289		
**Married marital status** († Single ^a^)	0.86 (0.71–1.05)	0.144		
**Partner not working outside home** († working partner)	1.05 (0.85–1.30)	0.683		
**Working partner profession** († non-healthcare)				
Physician	1.02 (0.84–1.24)	0.857		
Health care provider, non-physician	0.93 (0.71–1.23)	0.585		
**Not having children** († parents)	1.22 (0.97–1.57)	0.101		
**Children number**	1.00 (0.94–1.06)	0.936		
**Chronic disease history** († absent)	1.20 (1.03–1.39)	0.019*	1.12 (0.98–1.28)	0.112
**Previous depression or anxiety diagnosis** († absent)	1.08 (0.89–1.32)	0.425		
**Physical exercise frequency** († ≤Monthly)				
Weekly	0.96 (0.81–1.16)	0.676		
Daily	0.95 (0.73–1.26)	0.716		
**Occupation Specialist** († family physician)	1.25 (1.04–1.51)	0.021*	1.29 (1.09–1.54)	0.003*
**Rural practice Location** († urban)	1.30 (1.10–1.52)	0.002*	1.20 (1.03–1.40)	0.025*
**Part-time Employment status** († full-time)	1.17 (0.82–1.59)	0.321		
**Highest degree** († Bachelor)				
Diploma	1.08 (0.88–1.33)	0.463		
Master’s degree and higher	1.07 (0.89–1.30)	0.456		
**Managerial Position** († no)	0.94 (0.78–1.16)	0.577		
**Years in practice**	0.99 (0.99–1.00)	0.175		
**Ride time to work** in minutes ^b^	1.00 (0.99–1.00)	0.270		
**Weekend work ≥ 2/month** († ˂ 2/month)	1.15 (0.98–1.35)	0.098		
**Patient No. /shift**	1.00 (0.99–1.01)	0.089		
**High Professional fulfillment** († low)	0.84 (0.64–1.10)	0.189	0.80 (0.62–1.04)	0.102
**Burned out** († absent)	1.05 (0.91–1.22)	0.512	1.09 (0.94–1.27)	0.264
**Depression symptoms** († absent)	0.99 (0.85–1.16)	0.901	1.03 (0.87–1.21)	0.751
**Thoughts of leaving the profession** († Never / Rarely**)**				
Sometimes	0.82 (0.69–0.98)	0.032*	0.85 (0.72–1.00)	0.052
Often	1.03 (0.86–1.23)	0.755	0.96 (0.80–1.15)	0.671

†Reference Group.

*Bold fonts indicate statistically significant associations with *p*-value < 0.05.

^a^Single in marital status included single, separated, divorced, and widowed.

^b^Natural logarithm had been applied to handle non-linearity.

**Fig. 5:**
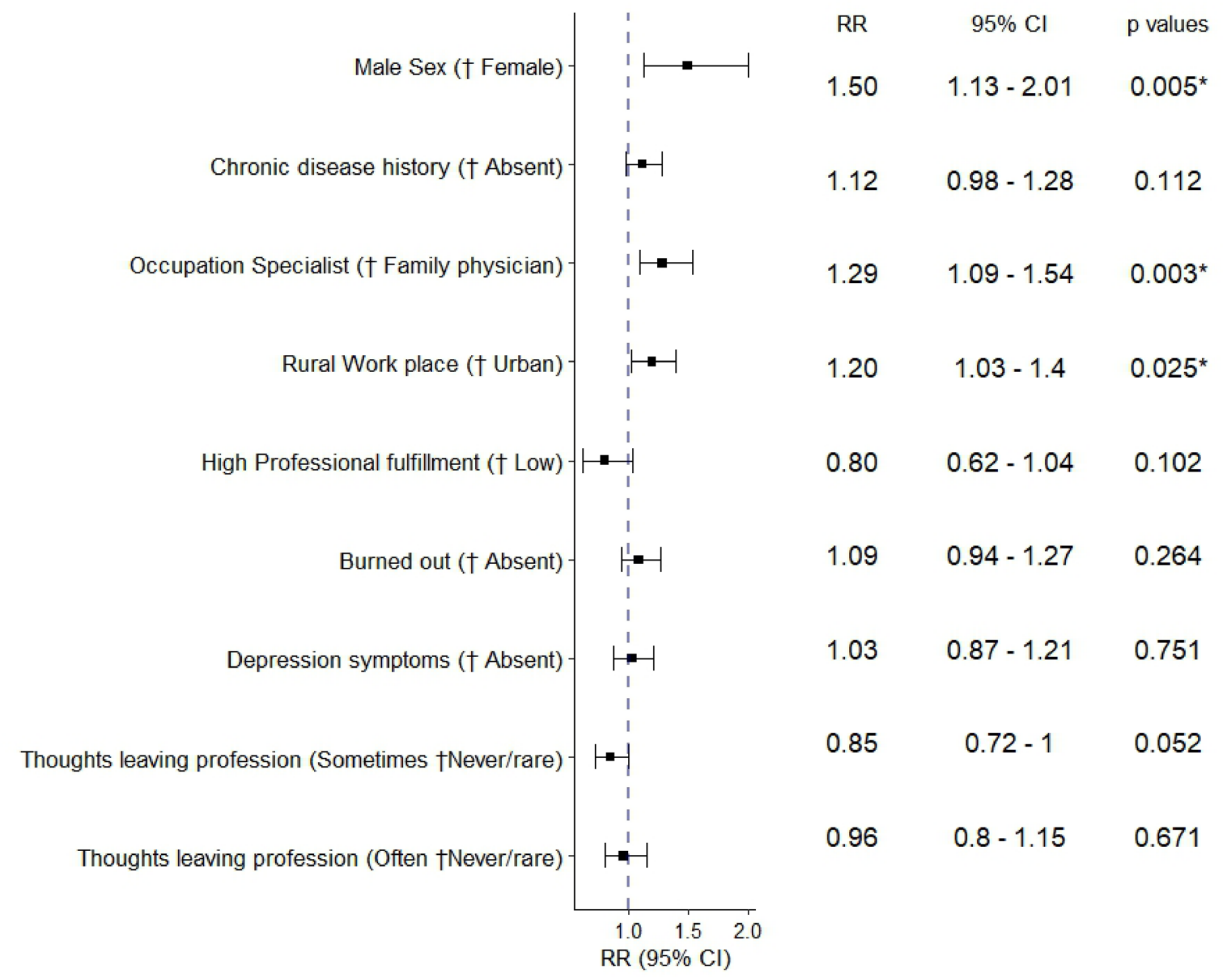
Factors related to prescribing errors in primary care settings in Alexandria, Egypt, 2022–2023: a Multivariate Negative Binomial Analysis. RR, rate ratio; CI, confidence interval. *Statistically significant associations with a *p*-value < 0.05. † Reference Group.

Only being male, occupation specialist, and working in rural settings were significantly associated with the error rates in the multivariate analysis (Fig. 5). The prescribing error rate for male physicians was 1.5 times greater than for female ones (95% CI 1.13–2.01; *p* = 0.005). Significantly higher error rates were linked to specialist occupation, compared to a family physician position with RR 1.29 (95% CI 1.09–1.54; *p* = 0.003). Rural employees had a 20% higher risk of medication errors than urban workers (RR, 1.20, 95% CI 1.03–1.40; *p* = 0.025). Depression symptoms were not associated with a higher risk of medication error rate (RR, 1.03, 95% CI, 0.87–1.21; *p* = 0.751). Similarly, burnout, low professional fulfillment, and quitting thoughts were not significant risk factors for prescribing errors.

## Discussion

This multicentric study of family physicians identified that most participants had low professional fulfillment, while almost half of them reported depression and burnout symptoms. The prescribing error rate was 46.34%, but only 0.11% represented serious potential patient harm. The most prevalent ordering errors were incorrect dosage regimens, incomplete patient data, and uncompleted prescribing information. No significant association was detected between depression, burnout, and low professional fulfillment and objectively evaluated prescribing errors. Significant risk factors for prescribing errors included male gender, specialist occupation, and rural work settings.

High burnout prevalence in the current study confirmed the alarming rates among primary care physicians, comparable levels in the United States (35%) and Japan (34%) emphasize the global burden of the problem^44,45^. Although lower burnout rates were reported in Saudi Arabia (25.2%) and Qatar (12.6%)^46,47^, The heterogeneity in the measurement instruments and burnout criteria might explain the variability among the studies, especially with the high frequency in burnout subcategories^48^. The staff shortage, elderly population, elevated medical demands, challenges in providing appropriate patient care, and financial constraints increase primary care physicians’ risk of burnout^49^.

Professional fulfillment was inversely linked to physicians’ quitting intentions^50^. Turnover of physicians entails productivity loss, recruitment, and training costs for organizations^51^. Therefore, the low rate of professional fulfillment in the current study raises a serious alarm, particularly as it aligns with earlier research among Egyptian physicians^52,53^. Similarly, a survey of 211 primary care doctors in Bahrain revealed an 82.6% low personal achievement rate^54^.

Depression is one of the prevalent mental illnesses among clinicians^55^. There is conflicting evidence regarding the association between burnout and depression in physicians^56^. The current findings highlighted the comparable rates between the two phenomena in the sample. depression prevalence in the current study lines up with the national research of 15,243 emergency doctors in China, in which 35.59% of the participants suffered from depression^57^. A meta-analysis study among physicians concluded a lower pooled global prevalence of depression (20.5%), but the study was limited by moderate bias risk and heterogeneity across included studies^58^.

Medication errors are responsible for more than 50% of all preventable injuries in health care worldwide, with an annual cost of 4.5–21.8 billion euros in Europe^10^. Prescribing errors occur frequently in outpatient and ambulatory healthcare facilities^59^. The present study’s ordering error rate supported the previous finding in different countries as a Bahraini primary healthcare department (44.3% of the prescribed drugs) and Malaysian primary care clinics (48.0% of prescriptions)^60,61^. Misagh et al. reported prescribing errors in each of the studied 2000 outpatient prescriptions in Iran^62^.

Literature studying prescribing errors shows variability in definitions, rate measuring methods, handwritten or electronic prescriptions, investigated population, and evaluation methods^63,64^. Medication error rate is influenced by the denominator of measurement, such as total prescription, prescribed item, or patient^65^. The global range of prescribing errors in outpatient settings was 0–91% of the total number of prescribed medications^59^. Investigations from Bahrain, China, and Saudi Arabia revealed a lower prescribing error rate (34.1% of prescriptions, 4.66% of prescriptions, and 0.09% of prescribed items respectively)^66–68^.

Most errors did not seriously threaten patients. In general practices in England, Avery et al. reported severe errors in 0.18% of the studied prescriptions^69^. Compared to actually measured harm, a recent systematic review also reported that the potential harm of medication errors in primary care is overestimated since they rarely cause emergency department visits or hospitalizations^70^.

The most prevalent prescribing error type in our study was the wrong dosage regimen. It was consistent with Abanmy et al.'s findings that incorrect doses accounted for 57.2% of prescribing errors in a Saudi family medicine setting^67^. Our results aligned with previous systematic reviews in outpatient settings and hospitals^59,71^.

Incomplete drug orders could jeopardize the quality of patient care by causing time loss, prescription misinterpretation, and improper medicine supply and administration^72^. Over 36% of prescribing error rates in the current study were writing process errors. Similarly, a high prevalence of incomplete information was observed in the Saudi hospital settings (25%) as well as the English general practice (26.1%)^73,74^. However, the current study supports previous findings that decision errors are more likely to threaten patient health compared to writing process errors^75^.

Electronic prescribing systems provided a successful solution to illegible handwriting and incomplete prescription information^76^. Despite links between electronic prescribing and fewer medication errors and adverse drug events, better patient safety, and time and cost savings, several concerns were raised about e-prescribing adoption. Electronic prescribing systems’ challenges include novel types of errors, implementation and maintenance expenses, team training, healthcare workers’ additional duties, prescribing software systems’ variability, and technical problems^77^. In addition, pharmacist intervention and physician education can help minimize prescribing errors^78^.

The current study’s top 3 inappropriately prescribed categories were consistent with the most prescribed drugs in outpatient settings in both India and Nepal^79,80^. Comparable results, in a different order and frequency, were observed in an investigation in Jordan, in which antibiotics accounted for 31.7% of prescribing errors, followed by analgesics (26.7%), and antihistamines (10.9%)^81^. However, the investigated family units used a local essential drug list, making it difficult to compare the specific drug data with prior literature.

Depression has a well-established association with burnout, whereas previous studies detected depressive symptoms as a significant predictor of medical errors independent of burnout^19,20,82^. Compared to non-depressed residents, depressed physicians committed significantly higher rates of medication errors on active surveillance^83^. In contrast, the rate of total medication error was unrelated to depression symptoms in the current study. A cohort study of 537 residents can explain this disparity since depression showed a significant association with harmful errors but not total medical errors^18^.

Using objective measures of prescribing errors, the current findings did not support the established association between physicians’ burnout symptoms and self-perceived medical errors in the literature even after controlling the depression presence^84^. The present findings are consistent with research that evaluated burnout’s association with physicians’ performance using medical charts and orders. Hewitt et al. reported no significant associations between residents’ mental health and objectively evaluated surgical outcomes, despite that burned-out residents are nearly 3 times more likely to self-report harmful medical errors^17^. Burnout did not significantly impact medical errors collected by independent research assistants among 31 French intensive care units^19^. Using active surveillance, Fahrenkopf et al. identified no significant difference in the rate of medication error between burnt-out and non-burnt-out residents^83^. According to a cohort study among American and Canadian resident physicians, burnout symptoms were unrelated to objectively detected medical errors^18^.

The inconsistent association between burnout and self-reported and observed medical errors may be attributed to physicians’ imprecise self-assessment of their performance^85^. Burned-out medical professionals are more prone to self-criticism, and hence more likely to disclose errors^16^. In addition, self-reported data are susceptible to social desirability and recall bias^86^.

Our findings indicated that errors were more likely to occur with male physicians. These outcomes were consistent with Assiri et al.'s cohort research in Saudi family medicine facilities. The research indicated that male physicians had a 1.6 times higher risk of committing PE than female physicians^87^. female clinicians were more likely to deliver preventative care, counsel patients, and comply with clinical guidelines than their male colleagues^88^.

Compared to consultants of different specialties, family physicians had a significantly lower rate of prescribing errors in the current study. Jayaweera et al. also indicated that general internists were more likely than family physicians to prescribe potentially inappropriate drugs^89^. Although the previous finding lacks a clear explanation, Tonelli et al.'s finding of significant variance in the complexity of patients examined by various medical professions may provide some insight. They identified family physicians’ patients as having the least complexity out of 13 medical specialties^90^.

According to the present study, there was a significant association between rural work environments and prescribing errors. Research in ambulatory care settings has also reported that rurality increases the risk of inappropriate prescriptions of antibiotics and geriatric medications^91,92^. With a growing elderly population and health demands coupled with a shrinking health system efficiency and resources, WHO identified primary care disparities among rural and urban regions as a global health threat^93^.

To the best of our knowledge, this study could be considered the first to investigate the association between objectively measured prescribing errors and burnout among primary care physicians in the region. A further strength of the study was its multicenter design, which involved 25 primary healthcare units. Lastly, the researchers who investigated prescribing errors were unaware of the participants’ levels of burnout and depression, and the study’s specific hypothesis was not disclosed to the participants, so there was no risk of change in the prescribing pattern.

On the other hand, certain limitations need to be considered when interpreting the study results. Firstly, a cross-sectional study design cannot establish causality. Secondly, the study reviewed only essential drug list medications in family health units, which may be misleading due to their limited variety. Thirdly, while two qualified pharmacists identified potential harm from errors, patients were not monitored for actual injuries. Fourthly, prescription errors lack data on inter-rater reliability. Fifthly, the analysis included only physicians who voluntarily agreed to participate in the study, and the majority of participants were females. Although this reflects the current situation in primary healthcare units participating in the study, the nonrandom sample may limit the findings’ generalizability.

## Conclusion

The alarming rates of burnout and depression in doctors highlight the necessity of understanding and handling these problems. Although prescribing errors were widespread in primary care settings, physician burnout and depression weren’t linked to them. Significant risk factors for prescribing errors were identified as male physicians, rural work locations, and non-family physician specialists. Longitudinal studies are necessary to evaluate the impact of physician burnout on objective measures of patient safety and enhance prescribing practices in ambulatory care settings.

## Supplementary Information

Below is the link to the electronic supplementary material.

Supplementary Material 1

## Data Availability

The study’s dataset can be obtained from the corresponding author upon request.

## Abbreviations

ATC: Anatomical therapeutic chemical
BNF: British national formulary
CDC: Centers for disease control
CI: Confidence interval
FHU: Family health units
ICD: International classification of diseases
IQR: Interquartile range
Mdn: Median
NICE: National institute for healthcare and excellence
PE: Prescribing errors
PFI: Professional fulfillment index
PHC: Primary health care
PHQ: Patient health questionnaire
PRN: Prescribing when needed
RR: Rate ratio
SPSS: Statistical package for the social sciences
WHO: World health organization

## References

[1] World Health Organization. Burn-out an “occupational phenomenon”: International Classification of Diseases: WHO; 2019 [Available from: https://www.who.int/news/item/28-05-2019-burn-out-an-occupational-phenomenon-international-classification-of-diseases.

[2] Shanafelt, T. D. et al. Changes in burnout and satisfaction with work-life integration in physicians and the general US working population between 2011 and 2020. *Mayo Clin. Proc.* **97**(3), 491–506 (2022). 35246286 10.1016/j.mayocp.2021.11.021

[3] Lancet, T. Physician burnout: The need to rehumanise health systems. *Lancet* **394**(10209), 1591 (2019). 31690430 10.1016/S0140-6736(19)32669-8

[4] Doraiswamy, S., Chaabna, K., Jithesh, A., Mamtani, R. & Cheema, S. Physician burnout in the Eastern Mediterranean region: influence of gender and related factors—Systematic review and meta-analysis. *J. Glob Health.* **11**, 04043 (2021). 34326993 10.7189/jogh.11.04043 PMC8285757

[5] Shanafelt, T.D., West, C.P., Dyrbye, L.N., Trockel, M., Tutty, M., Wang, H., et al. Changes in burnout and satisfaction with work-life integration in physicians during the first 2 years of the COVID-19 pandemic. (1942–5546 (Electronic)) (2022). 10.1016/j.mayocp.2022.09.002 PMC9472795 36229269

[6] West, C. P., Dyrbye, L. N. & Shanafelt, T. D. Physician burnout: Contributors, consequences and solutions. *J. Intern. Med.* **283**(6), 516–529 (2018). 29505159 10.1111/joim.12752

[7] Patel, R. S., Bachu, R., Adikey, A., Malik, M. & Shah, M. Factors related to physician burnout and its consequences: A review. *Behav. Sci. (Basel).* **8**(11), 98 (2018). 30366419 10.3390/bs8110098 PMC6262585

[8] Medication Without Harm - Global Patient Safety Challenge on Medication Safety [Internet]. 2017. Available from: https://iris.who.int/bitstream/handle/10665/255263/WHO-HIS-SDS-2017.6-eng.pdf. 10.1016/S0140-6736(17)31047-4 28463129

[9] Makary, M.A., & Daniel, M. Medical error-the third leading cause of death in the US. (1756–1833 (Electronic)) (2016). 10.1136/bmj.i2139 27143499

[10] Hodkinson, A. et al. Preventable medication harm across health care settings: A systematic review and meta-analysis. *BMC Med.* **18**(1), 313 (2020). 33153451 10.1186/s12916-020-01774-9 PMC7646069

[11] Shehata, Z. H. A., Sabri, N. A. & Elmelegy, A. A. Descriptive analysis of medication errors reported to the Egyptian national online reporting system during six months. *J. Am. Med. Inform. Assoc.* **23**(2), 366–374 (2015). 26254479 10.1093/jamia/ocv096 PMC11740539

[12] Watanabe, J. H., McInnis, T. & Hirsch, J. D. Cost of prescription drug-related morbidity and mortality. *Ann. Pharmacother.* **52**(9), 829–837 (2018). 29577766 10.1177/1060028018765159

[13] Mangory, K. Y., Ali, L. Y., Rø, K. I. & Tyssen, R. Effect of burnout among physicians on observed adverse patient outcomes: A literature review. *BMC Health Serv. Res.* **21**(1), 369 (2021). 33879135 10.1186/s12913-021-06371-x PMC8057942

[14] McTaggart, L. S. & Walker, J. P. The relationship between resident physician burnout and its’ effects on patient care, professionalism, and academic achievement: A review of the literature. *Health Sci. Rev.* **4**, 100049 (2022).

[15] Rathert, C., Williams, E. S. & Linhart, H. Evidence for the quadruple aim: A systematic review of the literature on physician burnout and patient outcomes. *Med. Care.* **56**(12), 976–984 (2018). 30339573 10.1097/MLR.0000000000000999

[16] Lawson, N. D. Burnout is not associated with increased medical errors. *Mayo Clin. Proc.* **93**(11), 1683 (2018). 30392547 10.1016/j.mayocp.2018.08.015

[17] Hewitt, D. B. et al. Association of surgical resident wellness with medical errors and patient outcomes. *Ann. Surg.* **274**(2), 396–402 (2021). 32282379 10.1097/SLA.0000000000003909

[18] Brunsberg, K.A., Landrigan Cp Fau – Garcia, B.M., Garcia Bm Fau – Petty, C.R., Petty Cr Fau – Sectish, T.C., Sectish Tc Fau – Simpkin, A.L., Simpkin Al Fau – Spector, N.D., et al. Association of Pediatric Resident Physician Depression and Burnout With Harmful Medical Errors on Inpatient Services. 2019(1938–808X (Electronic)). 10.1097/ACM.0000000000002778 PMC6667283 31045601

[19] Garrouste-Orgeas, M. et al. The Iatroref study: Medical errors are associated with symptoms of depression in ICU staff but not burnout or safety culture. *Intensive Care Med.* **41**(2), 273–284 (2015). 25576157 10.1007/s00134-014-3601-4

[20] Pereira-Lima, K. et al. Association between physician depressive symptoms and medical errors: A systematic review and meta-analysis. *JAMA Netw. Open.* **2**(11), e1916097-e (2019). 31774520 10.1001/jamanetworkopen.2019.16097 PMC6902829

[21] Koutsimani, P., Montgomery, A. & Georganta, K. The relationship between burnout, depression, and anxiety: A systematic review and meta-analysis. *Front. Psychol.* **10**, 284 (2019). 30918490 10.3389/fpsyg.2019.00284 PMC6424886

[22] World Health Organization. Patient safety. Global action on patient safety. who.int (2019).

[23] Organization, WH. Global burden of preventable medication-related harm in health care: a systematic review (2023).

[24] Egypt in numbers - population [Internet]. 2024 [cited 11/12/2024]. Available from: https://www.capmas.gov.eg/Pages/Publications.aspx?page_id=5104&Year=23602.

[25] Kamel, L., Dowara, S., & Eid, N. *Practice Guidelines for Family Physicians, 6 Volumes: Ministry of Health* (2007).

[26] Central agency for public mobilization & statistics ARoE. Annual bulletin of health services statistics at treatment at state’s expence at home and board 2021 (2023).

[27] Al-Haddad, A., Al-Omar, F., Al-Khaleel, A. & Al-Khalaf, A. Prevalence of burnout syndrome and its related risk factors among physicians working in primary health care centers of the Ministry of Health, Al Ahsa region, Saudi Arabia, 2018–2019. *J. Fam. Med. Prim. Care.* **9**(2), 571–579 (2020). 10.4103/jfmpc.jfmpc_743_19 PMC7113941 32318384

[28] Tawfik, D. S. et al. Physician burnout, well-being, and work unit safety grades in relationship to reported medical errors. *Mayo Clin. Proc.* **93**(11), 1571–1580 (2018). 30001832 10.1016/j.mayocp.2018.05.014 PMC6258067

[29] Dean, A.G., Arner, T.G., Sunki, G.G., Friedman, R., Lantinga, M., Sangam, S., et al. Epi Info™, a database and statistics program for public health professionals. CDC, Atlanta, GA, USA (2011).

[30] Kroenke, K., Spitzer, R. L. & Williams, J. B. The PHQ-9: Validity of a brief depression severity measure. *J. Gen. Intern. Med.* **16**(9), 606–613 (2001). 11556941 10.1046/j.1525-1497.2001.016009606.x PMC1495268

[31] Trockel, M. et al. A Brief instrument to assess both burnout and professional fulfillment in physicians: Reliability and validity, including correlation with self-reported medical errors, in a sample of resident and practicing Physicians. *Acad. Psychiatry.* **42**(1), 11–24 (2018). 29196982 10.1007/s40596-017-0849-3 PMC5794850

[32] Vilendrer, S. M. et al. How feedback is given matters: A cross-sectional survey of patient satisfaction feedback delivery and physician well-being. *Mayo Clin. Proc.* **96**(10), 2615–2627 (2021). 34479736 10.1016/j.mayocp.2021.03.039

[33] Alkhamees, A.A.-O., Assiri, H., Alharbi, H.Y., Nasser, A., & Alkhamees, M.A. Burnout and depression among psychiatry residents during COVID-19 pandemic. (1478–4491 (Electronic)) (2021). 10.1186/s12960-021-00584-1 PMC8022305 33823857

[34] Dean, B., Barber, N. & Schachter, M. What is a prescribing error?. *Qual. Health Care.* **9**(4), 232–237 (2000). 11101708 10.1136/qhc.9.4.232 PMC1743540

[35] Ghaleb, M. A., Barber, N., Dean Franklin, B. & Wong, I. C. What constitutes a prescribing error in paediatrics?. *Qual. Saf. Health Care.* **14**(5), 352–357 (2005). 16195569 10.1136/qshc.2005.013797 PMC1744084

[36] Joint Formulary Committee. BNF 85 (British National Formulary): March-September 2023: BMJ Group and the Royal Pharmaceutical Society of Great Britain (2023).

[37] Yang, J.-h., Liao, Y.-f., Lin, W.-b., & Wu, W. Prescribing errors in electronic prescriptions for outpatients intercepted by pharmacists and the impact of prescribing workload on error rate in a Chinese tertiary-care women and children’s hospital. BMC Health Services Research (2019). 10.1186/s12913-019-4843-1 PMC6936080 31888758

[38] Gates, P. J., Baysari, M. T., Mumford, V., Raban, M. Z. & Westbrook, J. I. Standardising the classification of harm associated with medication errors: The harm associated with medication error classification (HAMEC). *Drug. Saf.* **42**(8), 931–939 (2019). 31016678 10.1007/s40264-019-00823-4 PMC6647434

[39] World Health Organization (WHO). The Anatomical Therapeutic Chemical Classification System with Defined Daily Doses: World Health Organization; [Available from: https://www.who.int/standards/classifications/other-classifications/the-anatomical-therapeutic-chemical-classification-system-with-defined-daily-doses.

[40] Thomas, B. et al. Investigating the incidence, nature, severity and potential causality of medication errors in hospital settings in Qatar. *Int. J. Clin. Pharm.* **43**(1), 77–84 (2021). 32767219 10.1007/s11096-020-01108-y PMC7878234

[41] Mishra, P. et al. Descriptive statistics and normality tests for statistical data. *Ann. Card Anaesth.* **22**(1), 67–72 (2019). 30648682 10.4103/aca.ACA_157_18 PMC6350423

[42] IBM Corp. IBM SPSS Statistics for Windows ,Version 28.0. Armonk, NY: IBM Corp (2021).

[43] R Core Team. R: A language and environment for statistical computing. R Foundation for Statistical Computing, Vienna, Austria (2023).

[44] Hoff, T., Trovato, K. & Kitsakos, A. Burnout among family physicians in the United States: A review of the literature. *Qual. Manag. Health Care.* **33**(1), 1–11 (2023). 37817317 10.1097/QMH.0000000000000439

[45] Nonaka, S. et al. Prevalence of Burnout among Internal Medicine and Primary Care Physicians before and during the COVID-19 Pandemic in Japan. *Intern. Med.* **61**(5), 647–651 (2022). 34924459 10.2169/internalmedicine.8118-21 PMC8943365

[46] Abdulla, L., Al-Qahtani, D. M. & Al-Kuwari, M. G. Prevalence and determinants of burnout syndrome among primary healthcare physicians in Qatar. *S. Afr. Family Pract.* **53**, 380–383 (2011).

[47] Alshreem, R. M., Baraja, M. & Almogbel, E. S. Prevalence of burnout and its impact on self-reported patient care among primary health care physicians at King Abdul-Aziz Medical City in Riyadh region. *J. Family Med Prim. Care.* **11**(8), 4624–4630 (2022). 36352917 10.4103/jfmpc.jfmpc_1622_21 PMC9638661

[48] Rotenstein, L. S. et al. Prevalence of burnout among physicians: A systematic review. *JAMA* **320**(11), 1131–1150 (2018). 30326495 10.1001/jama.2018.12777 PMC6233645

[49] Gerteis, J., Booker, C., Brach, C., & De La Mare, J. *Burnout in Primary Care: Assessing and Addressing It in Your Practice*. Rockville, MD: Agency for Healthcare Research and Quality (2023).

[50] Collins, R. T., Schadler, A., Huang, H., Day, S. B. & Bauer, J. A. Impact of burnout and professional fulfillment on intent to leave among pediatric physicians: The findings of a quality improvement initiative. *BMC Health Serv. Res.* **24**(1), 434 (2024). 38580940 10.1186/s12913-024-10842-2 PMC10998309

[51] Williams, E.A.-O., Rathert, C., & Buttigieg, S.C. The personal and professional consequences of physician burnout: A systematic review of the literature. 2019(1552–6801 (Electronic)). 10.1177/1077558719856787 31216940

[52] Abdelhafiz, A. S. et al. Prevalence, associated factors, and consequences of burnout among Egyptian physicians during COVID-19 pandemic. *Front. Public Health.* **8**, 590190 (2020). 33344401 10.3389/fpubh.2020.590190 PMC7744472

[53] Shaltout, A. Prevalence of burnout syndrome among working physicians in family health centres and units in port said governorate. *Asian J. Med. Health.* **21**, 25–43 (2023).

[54] Ubaidi, B.A.A., Helal, S., Al-Eid, K., Rashid, L., Al-Showaiter, H., AlAsheeri, K., et al., (ed). *A Study on the Prevalence of Burnout Among Primary Care Physicians on the Kingdom of Bahrain* (2020).

[55] Baker, K., Warren, R., Abelson, J. L. & Sen, S. Physician mental health: Depression and anxiety. In *Physician Mental Health and Well-Being: Research and Practice* (eds Brower, K. J. & Riba, M. B.) 131–150 (Springer, 2017).

[56] Bianchi, R. et al. Is burnout a depressive condition? A 14-sample meta-analytic and bifactor analytic study. *Clin. Psychol. Sci.* **9**(4), 579–597 (2021).

[57] Chen, Y. et al. Prevalence and predictors of depression among emergency physicians: A national cross-sectional study. *BMC Psychiatry* **22**(1), 69 (2022). 35090424 10.1186/s12888-022-03687-8 PMC8795725

[58] Johns, G., Samuel, V., Freemantle, L., Lewis, J. & Waddington, L. The global prevalence of depression and anxiety among doctors during the covid-19 pandemic: Systematic review and meta-analysis. *J. Affect. Disord.* **298**(Pt A), 431–441 (2022). 34785264 10.1016/j.jad.2021.11.026 PMC8596335

[59] Naseralallah, L., Stewart, D., Price, M. & Paudyal, V. Prevalence, contributing factors, and interventions to reduce medication errors in outpatient and ambulatory settings: A systematic review. *Int. J. Clin. Pharm.* **45**(6), 1359–1377 (2023). 37682400 10.1007/s11096-023-01626-5 PMC10682158

[60] Aljasmi, F., Almalood, F. & Al, A. A. Prevalence of medication errors in primary health care at Bahrain Defence Force Hospital—Prescription-based study. *Drug. Healthc. Patient. Saf.* **10**, 1–7 (2018). 29445304 10.2147/DHPS.S147994 PMC5808686

[61] Lim, W.Y., Hss, A.S., Ng, L.M., John Jasudass, S.R., Sararaks, S., Vengadasalam, P., et al. The impact of a prescription review and prescriber feedback system on prescribing practices in primary care clinics: A cluster randomised trial (2018). 10.1186/s12875-018-0808-4 PMC6053727 30025534

[62] Misagh, P., Vazin, A. & Namazi, S. Evaluation of faculty and non-faculty Physicians’ medication errors in outpatients’ prescriptions in Shiraz, Iran. *Iran J. Pharm. Res.* **17**(Suppl), 151–158 (2018). 29796040 PMC5958335

[63] Albarrak, A. I., Al Rashidi, E. A., Fatani, R. K., Al Ageel, S. I. & Mohammed, R. Assessment of legibility and completeness of handwritten and electronic prescriptions. *Saudi Pharm. J.* **22**(6), 522–527 (2014). 25561864 10.1016/j.jsps.2014.02.013 PMC4281619

[64] Ghadah Asaad, A. et al. What is the epidemiology of medication errors, error-related adverse events and risk factors for errors in adults managed in community care contexts? A systematic review of the international literature. *BMJ Open* **8**(5), e019101 (2018). 10.1136/bmjopen-2017-019101 PMC5942474 29730617

[65] World Health Organization. Medication errors: Technical series on safer primary care (2016).

[66] Al Khaja, K. A. J., Ahmed Isa, H., Veeramuthu, S. & Sequeira, R. P. Potentially inappropriate prescribing in older adults with hypertension or diabetes mellitus and hypertension in a primary care setting in Bahrain. *Med. Princ. Pract.* **27**(3), 241–249 (2018). 29495011 10.1159/000488055 PMC6062721

[67] Abanmy, N., Alrowibah, F., Al Juffali, L., Ebrahim, A. & Kofi, M. Prescribing errors among family and community medicine center in Riyadh, Saudi Arabia. *Afr. J. Pharm. Pharmacol* **14**, 163–168 (2020).

[68] Tan, L. et al. A survey of prescription errors in paediatric outpatients in multi-primary care settings: The implementation of an electronic pre-prescription system. *Front. Pediatr.* **10**, 880928 (2022). 35757118 10.3389/fped.2022.880928 PMC9218205

[69] Avery, A. J. et al. The prevalence and nature of prescribing and monitoring errors in English general practice: A retrospective case note review. *Br. J. Gen. Pract.* **63**(613), e543–e553 (2013). 23972195 10.3399/bjgp13X670679 PMC3722831

[70] Richard, A. Y. et al. Ambulatory medication safety in primary care: A systematic review. *J. Am. Board Family Med.* **35**(3), 610 (2022). 10.3122/jabfm.2022.03.210334 PMC9730343 35641040

[71] Thomas, B. et al. Medication errors in hospitals in the Middle East: A systematic review of prevalence, nature, severity and contributory factors. *Eur. J. Clin. Pharmacol.* **75**(9), 1269–1282 (2019). 31127338 10.1007/s00228-019-02689-y

[72] Velo, G. P. & Minuz, P. Medication errors: Prescribing faults and prescription errors. *Br. J. Clin. Pharmacol.* **67**(6), 624–628 (2009). 19594530 10.1111/j.1365-2125.2009.03425.x PMC2723200

[73] Nde-Eshimuni, S., Brian, G.B., Kate, M., Gill, G., Glen, S., Mindy, B., et al. The frequency and nature of prescribing problems by GPs-in-training (REVISiT): A retrospective review. *BJGP Open*. **6**(3):BJGPO.2021.0231 (2022). 10.3399/BJGPO.2021.0231 PMC9680734 35523432

[74] Alshahrani, S., Alakhali, K. & Al-Worafi, Y. Medication errors in a health care facility in southern Saudi Arabia. *Trop. J. Pharm. Res.* **18**, 1119–1122 (2019).

[75] Ather, A. et al. A study on determination of prescription writing errors in out patient department of medicine in a teaching Hospital. *Indian J. Pharm. Pract.* **6**(2), 21–24 (2013).

[76] Kenawy, A. S. & Kett, V. The impact of electronic prescription on reducing medication errors in an Egyptian outpatient clinic. *Int. J. Med. Informatics* **127**, 80–87 (2019). 10.1016/j.ijmedinf.2019.04.005 31128835

[77] Esmaeil Zadeh, P. & Tremblay, M. C. A review of the literature and proposed classification on e-prescribing: Functions, assimilation stages, benefits, concerns, and risks. *Res. Soc. Adm. Pharm.* **12**(1), 1–19 (2016). 10.1016/j.sapharm.2015.03.001 25847858

[78] Manias, E., Kusljic, S. & Wu, A. Interventions to reduce medication errors in adult medical and surgical settings: A systematic review. *Ther. Adv. Drug. Saf.* **11**, 2042098620968309 (2020). 33240478 10.1177/2042098620968309 PMC7672746

[79] Abidi, A., Gupta, S., Kansal, S. & Ramgopal, R. Prescription auditing and drug utilization pattern in a tertiary care teaching hospital of western UP. *Int. J. Basic Clin. Pharmacol.* **1**, 184–190 (2012).

[80] Shrestha, R. & Prajapati, S. Assessment of prescription pattern and prescription error in outpatient Department at Tertiary Care District Hospital. *Central. Nepal. J. Pharm. Policy Pract.* **12**, 16 (2019). 31321037 10.1186/s40545-019-0177-y PMC6617589

[81] Taleb, Y. A., Jaleel, S. A., Safi, M., Saeed, S., & Bdairat, M.A. Prescription errors and pharmacist intervention at outpatient pharmacy of prince Zaid Bin Al Hussien Hospital (RMS), Jordan. *Int. Res. J. Pharm. Med. Sci.*, pp. 11–13 (2019).

[82] Ryan, E., Hore, K., Power, J. & Jackson, T. The relationship between physician burnout and depression, anxiety, suicidality and substance Abuse: A mixed methods systematic review. *Front. Public Health.* **11**, 1133484 (2023). 37064688 10.3389/fpubh.2023.1133484 PMC10098100

[83] Fahrenkopf, A. M. et al. Rates of medication errors among depressed and burnt out residents: Prospective cohort study. *BMJ* **336**(7642), 488 (2008). 18258931 10.1136/bmj.39469.763218.BE PMC2258399

[84] Owoc, J., Mańczak, M., Jabłońska, M., Tombarkiewicz, M. & Olszewski, R. Association between physician burnout and self-reported errors: Meta-analysis. *J. Patient Saf.* **18**(1), e180–e188 (2022). 34951608 10.1097/PTS.0000000000000724

[85] Davis, D. A. et al. Accuracy of physician self-assessment compared with observed measures of competence: A systematic review. *JAMA* **296**(9), 1094–1102 (2006). 16954489 10.1001/jama.296.9.1094

[86] Althubaiti, A. Information bias in health research: Definition, pitfalls, and adjustment methods. *J. Multidiscip. Healthc.* **9**, 211–217 (2016). 27217764 10.2147/JMDH.S104807 PMC4862344

[87] Assiri, G. A., Alkhenizan, A. H., Al-Khani, S. A., Grant, L. M. & Sheikh, A. Investigating the epidemiology of medication errors in adults in community care settings. A retrospective cohort study in central Saudi Arabia. *Saudi Med. J.* **40**(2), 158–167 (2019). 30723861 10.15537/smj.2019.2.23933 PMC6402461

[88] Joseph, M. M., Ahasic, A. M., Clark, J. & Templeton, K. State of women in medicine: History, challenges, and the benefits of a diverse workforce. *Pediatrics* **148**(Supplement 2), e2021051440C (2021). 34470878 10.1542/peds.2021-051440C

[89] Jayaweera, A., Chung, Y. & Jabbarpour, Y. Primary care physician characteristics associated with prescribing potentially inappropriate medication for elderly patients: Medicare part D data. *J. Am. Board. Fam. Med.* **33**(4), 561–568 (2020). 32675267 10.3122/jabfm.2020.04.190310

[90] Tonelli, M. et al. Comparison of the complexity of patients seen by different medical subspecialists in a universal health care system. *JAMA Netw. Open.* **1**(7), e184852 (2018). 30646392 10.1001/jamanetworkopen.2018.4852 PMC6324421

[91] Nguyen, K., Subramanya, V. & Kulshreshtha, A. Risk factors associated with polypharmacy and potentially inappropriate medication use in ambulatory care among the elderly in the United States: A cross-sectional study. *Drugs-real World Outcomes* **10**(3), 357–362 (2023). 37233904 10.1007/s40801-023-00358-2 PMC10491561

[92] Yau, J. W., Thor, S. M., Tsai, D., Speare, T. & Rissel, C. Antimicrobial stewardship in rural and remote primary health care: A narrative review. *Antimicrob. Resist. Infect. Control* **10**(1), 105 (2021). 34256853 10.1186/s13756-021-00964-1 PMC8278763

[93] Organization, WH. Imbalances in rural primary care: A scoping literature review wih an emphasis on the WHO European Region, p. 64 (2018).

