# Supplementary material for: Burnout, depression, and medication errors among family physicians
Source: Sci Rep. 2025 Dec 9;15:43389. doi: 10.1038/s41598-025-29450-z (PMC12690096; doi:10.1038/s41598-025-29450-z)
Supplement: Supplementary file 1 — Supplementary Material 1 [file 41598_2025_29450_MOESM1_ESM.docx]

**Title:** **Burnout, depression, and medication errors among family physicians**

**Authors: Dina Fawzy Abd Elsadek, Amira B Kassem, Fayek ElKhwsky, Iman El Sayed**

***Corresponding author: Dina Fawzy Abd Elsadek**

**Affiliation:**

**Master’s Program, Department of Biomedical Informatics and Medical Statistics, Medical Research Institute, Alexandria University, Alexandria, Egypt**

**E-mail: mri.dina.m.informatics19@alexu.edu.eg**

**Amira B Kassem**

**Affiliation:**

**Department of clinical pharmacy and pharmacy practice, Faculty of Pharmacy, Damanhur University, Damanhur, Egypt.**

**E-mail: [Amira.Kassem@pharm.dmu.edu.eg](mailto:Amira.Kassem@pharm.dmu.edu.eg)**

**Fayek ElKhwsky**

**Affiliation:**

**Department of Biomedical Informatics and Medical Statistics, Medical Research Institute, Alexandria University, Alexandria, Egypt**

**E-mail: Felkhwsky@alexu.edu.eg**

**Iman El Sayed**

**Affiliation:**

**Department of Biomedical Informatics and Medical Statistics, Medical Research Institute, Alexandria University, Alexandria, Egypt**

**E-mail:** [**Eman.abd.elftaah@alexu.edu.eg**](mailto:Eman.abd.elftaah@alexu.edu.eg)

**ORCID 0000-0003-1088-958X**

**Supplementary Table S1:** **Examples of prescribing errors and potential harm**

| **Prescribing errors** | | **Example** | **Potential harm** |
| --- | --- | --- | --- |
| **Process ordering error** | | | |
| 1 | Incomplete or wrong patient information | Incomplete diagnosis | No harm |
| 2 | Wrong drug name | The physician wrote only “vitamin” with no specification | Minor |
| 3 | Incomplete prescribing instructions | The physician didn't write the duration for paracetamol. | Minor |
| **Decision ordering error** | | | |
| 1 | Drug without indication | Amoxicillin was written with the indication of a common cold | Moderate |
| 2 | Therapeutic duplication | Calcium supplements contain vitamin D3 500 IU/2.5 ml and Cholecalciferol oral drops 400 IU were prescribed at the same time to prevent vitamin D deficiency in a 5-month child | Minor |
| 3 | Indication without drug | The physician didn't initiate iron supplements for an anemic patient | Moderate |
| 4 | Drug contraindication | Tiemonium methyl sulphate was prescribed in infants younger than 6 months. | Moderate |
| 5 | Wrong route of administration | Metronidazole oral suspension was written for topical use in rosacea treatment | Minor |
| 6 | Drug interaction | The physician prescribed iron supplements and calcium carbonate once daily without time intervals. | Moderate |
| 7 | Wrong dose regimen (dose/ frequency) | Amlodipine 10 mg was prescribed twice daily which exceeds the maximum daily dose of 10 mg. | Serious |
| 8 | Wrong duration | Amoxicillin was prescribed for pharyngitis for 3 days instead of at least 5 days and 10 days is preferred. | Moderate |
| 9 | Wrong dilution instructions | Rehydration oral solution was prescribed as 100 ml after each diarrhea instead of dissolve on 200 ml and use 50-100 ml | Moderate |

**Supplementary Table S2:** **Top ten drug groups involved in the prescribing errors according to the ATC classification system**

| **ATC 2nd level** | **Involved drugs** | **Frequency** | **Percentage** |
| --- | --- | --- | --- |
| Analgesics | Paracetamol | 1309 | 20.01 |
| Antihistamines for systemic use | Chlorpheniramine maleate | 1155 | 17.65 |
| Antibacterials for systemic use | Amoxicillin, amoxicillin/clavulanic acid, amoxicillin/flucloxacillin, ampicillin/ sulbactam, benzathine penicillin, cefotaxime, metronidazole, and sulfamethoxazole/trimethoprim | 1122 | 17.15 |
| Antianemic preparations | Ferric hydroxide poly maltose complex, ferrous gluconate, and iron amino acid chelate plus folic acid | 495 | 7.57 |
| Cough and cold preparations | Ambroxol | 384 | 5.87 |
| Drugs for acid-related disorders | Omeprazole | 367 | 5.61 |
| Anti-inflammatory and antirheumatic products | Ibuprofen and diclofenac | 207 | 3.16 |
| Antibiotics and chemotherapeutics for dermatological use | Tetracycline, acyclovir, gentamicin, and silver sulfadiazine | 195 | 2.98 |
| Drugs for obstructive airway diseases | Salbutamol | 180 | 2.75 |
| Antifungals for dermatological use | Clotrimazole | 163 | 2.49 |
